# Gut microbial diversity impacts carbohydrate fermentation by children with severe acute malnutrition

**DOI:** 10.1016/j.isci.2026.114640

**Published:** 2026-01-07

**Authors:** Akshay Bisht, Jennifer Ahn-Jarvis, Kendall Corbin, Suzanne Harris, Perla Troncoso-Rey, Peter Olupot-Olupot, Nuala Calder, Kevin Walsh, Kathryn Maitland, Gary Frost, Frederick J. Warren

**Affiliations:** 1 Food, Microbiome and Health, Quadram Institute Bioscience, Norwich, UK; 2 Department of Horticulture, College of Agriculture, Food and Environment, University of Kentucky, Lexington, KY, USA; 3 London School of Hygiene and Tropical Medicine, Keppel Street, London, UK; 4 Mbale Clinical Research Institute, Busitema University Faculty of Health Sciences, Mbale Campus, Palissa Road, Mbale, Uganda; 5 Imperial Centre for Paediatrics and Child Health, Faculty of Medicine, Imperial College, London, UK; 6 Division of Diabetes, Endocrinology and Metabolism, Department of Medicine, Imperial College, London, UK; 7 Department of Nutritional Sciences, School of Life Course and Population Sciences, Faculty of Life Sciences and Medicine, King’s College, London, UK; 8 Institute of Global Health and Innovation, Faculty of Medicine, Imperial College, London, UK

**Keywords:** Microbiology, Microbiome, Pediatrics

## Abstract

African children suffering from severe acute malnutrition (SAM) have a disrupted gut microbiome and low short-chain fatty acids (SCFAs). These are linked to persistently high mortality and morbidity rates. Supplementing recovery feeding regimes with suitable fermentable carbohydrate may improve outcomes in SAM. We adapted *in vitro* colon models to investigate the ability of children with SAM to utilize four carbohydrate substrates: milk powders (with and without human milk-like oligosaccharides), chickpea-enriched feed, and inulin. All substrates, except inulin, were fermented to produce SCFAs. The inability to utilize inulin *ex vivo*, a widely used prebiotic, is attributed to low microbial diversity, enriched with Proteobacteria. Stool samples obtained after partial anthropometric recovery showed increased microbial diversity and higher levels of GH32 enzyme family, responsible for inulin metabolism. These findings can inform the design of future therapeutic feeds for the treatment of SAM, where inulin has been found ineffective during initial hospitalization. Alternative carbohydrates appear to be more effective in supporting gut recovery during both the initial and later treatment phases.

## Highlights

• Children with SAM have high fecal Proteobacteria, especially *Escherichia*-*Shigella*
• 7 days post-hospitalization, they cannot effectively utilize inulin *ex vivo*
• By 90 days post-care, Firmicutes increases and Proteobacteria decreases
• At 90 days, GH32 enzyme family levels rise, enabling inulin utilization and SCFA production

## Graphical abstract

**Figure:**
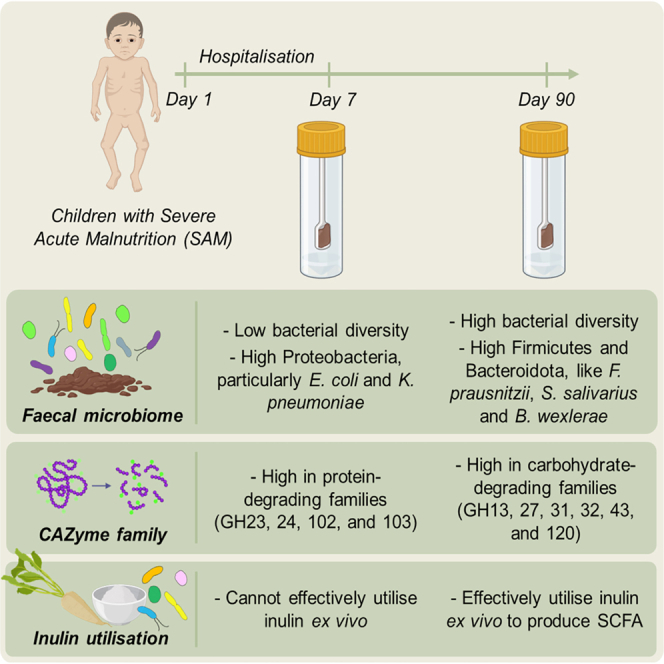

## Introduction

Childhood undernutrition remains a predominant problem in many lower- and middle-income countries, contributing to nearly half of all deaths in children under the age of 5 years.^1^^,^^2^ It is estimated that in 2022, 149 million children under the age of 5 years had stunted growth (height-for-age *Z* score ≤ −2), while 45 million exhibited wasting (weight-for-height *Z* score [WHZ] ≤ −2).^3^ In Africa, although less prevalent than stunting, the most severe form of wasting, severe acute malnutrition (SAM), is associated with high mortality and relapse rates.^4^ For children hospitalized with SAM, up to 20% die, with a high proportion of deaths (about 70%) occurring during the first week of hospitalization.^5^ Nutritional rehabilitation alongside supportive treatments has been the cornerstone of SAM treatment. The current World Health Organization (WHO) recommendation for nutritional treatment of children hospitalized with SAM is milk-based feeds (called F75 and F100, indicating their calorie content/100 mL) for inpatient management, followed by ready-to-use therapeutic feeds (RUTFs) for rehabilitation. This treatment regime leads to weight gain and recovery of appetite, as well as improvement in biochemical markers such as glycemic control.^6^ Despite this, nutritional recovery (along with recovery of anthropomorphic indicators) in African children hospitalized with SAM is a poor indicator of long-term outcomes, including increased risk of re-hospitalization with pneumonia or diarrhea or even death within a year following initial hospitalization,^7^^,^^8^ indicating that the period of vulnerability and damage sustained in SAM is far more complex and long-lasting than simple nutritional deficiencies.

Understanding of the human gut microbiota is exponentially advancing and has highlighted the role of the gut microbiota in influencing many physiological processes, including nutrient acquisition,^9^ growth hormone signaling and appetite control,^10^ and immune regulation,^11^ mediated through short-chain fatty acid (SCFA) production. During recovery from SAM, there is evidence of systematic dysregulation of many of these processes, leading to altered appetite regulation, impaired acquisition of key nutrients, and increased susceptibility to a range of infections.^12^ This points to the potential for a key role for the microbiota in the pathogenesis and rehabilitation of SAM.

The studies of the fecal microbiota of children suffering from SAM have revealed dramatic alterations in the gut microbiota.^13^^,^^14^^,^^15^^,^^16^^,^^17^^,^^18^^,^^19^ Twin studies on infants discordant for SAM have demonstrated that distinct microbiota changes occur as a result of SAM, independent of host genetics, environment, and background diet.^13^ These changes include a general reduction in bacterial diversity, as well as increases in potentially pathogenic bacteria such as *Klebsiella* and Enterobacteriaceae, and a reduction in beneficial saccharolytic bacteria in *Clostridium* clusters IV and XIVa (such as *Blautia*, Lachnospiraceae, Ruminococcaceae, and *Faecalibacterium prausnitzii*), *Bacteroides* species, and *Lactobacillus*.^15^^,^^18^^,^^20^ It has been hypothesized that some aspects of the microbiome of children with SAM represent a failure of the gut microbiota to fully mature,^15^^,^^16^ although there is also strong evidence that there is significant overgrowth of potential enteropathogens^17^^,^^20^ and risk of invasive bacterial infection.^5^

Emerging evidence indicates that reversing the gut microbiota changes that occur as a result of SAM has the potential to improve outcomes,^21^ although gut microbiome-targeted therapies have to be carefully designed, as interventions using probiotics and synbiotics have had limited success.^20^^,^^22^ The most promising approach is the use of the so-called microbiota-directed foods,^21^^,^^23^^,^^24^ in which food ingredients rich in carbohydrates that are fermentable by the gut microbiota of children with malnutrition are supplemented to conventional RUTFs. However, the majority of studies examining microbiota-directed foods are conducted in low-risk groups (mild to moderate malnutrition) in the community setting, where malnourished children will have better gut health and fewer comorbidities than children being treated for SAM in a hospital setting. Recently, two trials examining legume-based feeds (modifying gut integrity and microbiome using legume-based feeds [MIMBLE]) in children hospitalized with SAM have demonstrated that using suitable fermentable carbohydrate is crucial to address the damage to the gut microbiota diversity (which occurs during SAM) and improve clinical outcomes.^25^^,^^26^ The damage to the gut microbiome during SAM has the potential to restrict the range of substrates that are accessible to the microbiota. Therefore, by successfully identifying substrates that support the metabolism of the broadest range of microbial species possible, an increase in SCFAs may be achieved.

In this study, we aimed to determine the fermentability of potential microbiota-directed foods using an *in vitro* batch fermentation model inoculated with fecal samples collected from children with SAM on day 7 post-hospitalization. We investigated the fermentation of 4 different substrates: (1) infant formula (IF)—to represent the current WHO recommended recovery protocol of F75/F100 formula; (2) IF enriched with human milk-like oligosaccharide (HMO) (2′-fucosyllactose [2′-FL], IF + HMO)—to mimic human milk; (3) inulin, a fructan oligosaccharide—a widely used prebiotic carbohydrate; and (4) chickpea-enriched feed high in resistant starch specifically designed for use in intervention trials to support microbial recovery in children with SAM (MIMBLE).^25^^,^^26^ We then aimed to develop a mechanistic understanding of inulin fermentation by investigating its utilization at different SAM treatment stages. We implemented a paired study design to examine the *in vitro* fermentability of inulin in the presence of fecal samples collected from children with SAM on days 7 and 90 post-hospitalization (representing sick and partially anthropometrically recovered cohorts, respectively). During fermentation, changes in the bacterial community and SCFA production were monitored. It must be noted that children with SAM have markedly impaired nutrient absorption due to villous atrophy and reduced digestive capacity; as a result, a large portion of ingested food reaches the lower gut for colonic fermentation.^27^^,^^28^^,^^29^ Therefore, in this study, we did not predigest the samples.

## Results

### Children with SAM have a high proportion of Proteobacteria

**Figure 1:**
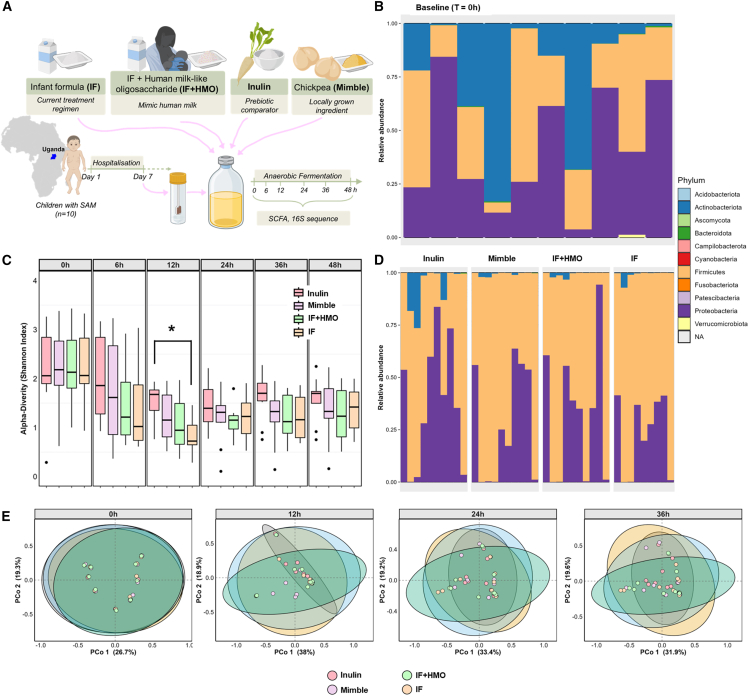
Changes in microbial profile in the presence of different substrates (A) Study design. The substrates tested were IF, IF + HMO, inulin, and chickpea-enriched feed (MIMBLE). Microbial composition was determined by 16S rRNA sequencing using QIIME2 coupled with phyloseq and phylosmith pipelines. (B–E) Phylogeny profile shows the phylum-level abundances of bacterial taxa for individual participants (*n* = 10) at (B) the start of the experiment (baseline, T = 0 h) and (D) after 36 h of *in vitro* fermentation in the presence of different substrates. (C and E) Changes in (C) alpha diversity (Shannon index) and (E) beta diversity (Bray-Curtis index) over time for each of the substrates during *in vitro* fermentation. Statistically significant differences are indicated with ∗*p* value <0.05; data are expressed as mean ± SEM.

The fecal samples were collected from children (*n* = 10) undergoing inpatient care with SAM at the Mbale Regional Referral Hospital, Uganda, between February and April 2016. SAM was defined according to WHO guidelines, using one or more of mid-upper arm circumference (MUAC) < 11.5 cm, WHZ < −3, or signs of nutritional edema.^6^ The average age of children was 2.2 years, ranging from 0.9 years to 4.5 years. All these children met the criteria for SAM with an average WHZ of −3.2 and MUAC of 11.1 cm and 6 had edema (Table 1). Fecal microbiome was analyzed using 16S rRNA sequencing, and large inter-individual differences in microbiome composition were observed (at baseline, T = 0 h, before start of fermentation) (Figure 1B). All samples contained a high proportion of Proteobacteria, particularly *Escherichia*-*Shigella*, which represents up to 75% of the total microbial abundance in some samples (Figure S1). In addition to Proteobacteria, the samples were dominated by bacteria from Actinobacteriota, a phylum that is primarily represented by the genus *Bifidobacterium* in the human gut, and Firmicutes, as would be expected in developing children microbiomes.^30^ Conversely, very low abundances of Bacteroidota were observed in these samples. The study cohort consisted predominantly of children with kwashiorkor-type malnutrition; therefore, caution is warranted when extending these findings to children with marasmic or marasmic-kwashiorkor types of malnutrition.

**Table 1:** Demographics of children receiving standard feeds on day 7 after hospitalization

Demographic	Total ( = 10)	Female (*n* = 5)	Male (*n* = 5)
**Parameters**			

Age (years)	2.2 ± 0.4	2.1 ± 0.6	2.4 ± 0.6
MUAC (cm)	11.1 ± 0.5	10. 4 ± 0.6	11.8 ± 0.8
Height (cm)	74.9 ± 4.0	71.2 ± 4.8	78.6 ± 6.6
Weight (kg)	7.3 ± 0.9	6.0 ± 0.8	8.7 ± 1.5
WHZ	−3.2 ± 0.5	−3.9 ± 0.6	−2.5 ± 0.8
Edema: ≥3 grade	6/10	3/5	3/5
Diarrhea	3/10	2/5	1/5
Breast feeding	1/10	0/5	1/5
Days receiving F75 feed up to day 7	5.0 ± 2.3	6.0 ± 2.2	4.0 ± 2.1
Days receiving F100 feed up to day 7	2.0 ± 2.3	1.0 ± 2.2	3.0 ± 2.1

**Clinical features**			

Pulse (beats/minute)	131 ± 6	133 ± 8	130 ± 8
Respiration rate (breaths/minute)	30 ± 1	31 ± 2	30 ± 1
Temperature (°C)	36.5 ± 0.2	36.8 ± 0.2	36.2 ± 0.2
Mean blood glucose over 72 h following admission (mmol/L)	5.2 ± 0.4	5.1 ± 0.6	5.4 ± 0.5

Data are presented as mean ± standard error of the mean.

Fecal samples from children were obtained on day 7 after hospitalization and were used for the screening study.

### Substrates drive differences in microbiome composition

Given the role of carbohydrates in driving changes in microbial composition, we sought to investigate the ability of children with SAM to utilize different carbohydrates as substrates during colonic fermentation. *In vitro* fermentation experiments were established using a batch colon fermentation system inoculated with fecal samples from children with SAM (*n* = 10) who were on standard WHO nutritional and supportive treatments. Fermentation was performed in the presence of IF, IF + HMO, inulin, and chickpea-enriched MIMBLE feed as substrates (nutritional composition provided in Table S1). Samples were taken at defined time points (with baseline samples at T = 0 h, taken before fermentation) (Figure 1A).

The microbial composition changed with the progression of *in vitro* fermentation, depending on both time and substrate (Figures 1C and 1D). Immediately following inoculation (at T = 0 h), the microbial community composition for each of the substrates was highly similar, as all samples clustered very closely together on a Principle Coordinate Analysis (PCoA) plot (Figure 1E), reflecting the individual microbial composition of each donor. Following sampling at subsequent time points, the microbiome compositions deviated and moved away from the composition at T = 0 h, although there were no significant differences (*p* > 0.05) observed between the substrates.

Across all the substrates, a reduction in microbial alpha diversity (Shannon index) was observed over time, although this did recover slightly at later time points (Figure 1C). The greatest reduction in microbial diversity was observed for IF and IF + HMO substrates at the 12 h time point. In contrast, the Shannon diversity in the presence of inulin was less reduced at 12 h and at subsequent time points compared to the other substrates. The decline in alpha diversity is driven by selective pressures imposed by the fermentation of defined substrates and is indicative of active fermentation. The changes in microbial diversity are reflected in the phylogeny profiles (Figure 1D), where the inulin remains similar to baseline after 36 h fermentation, whereas the other three substrates lead to a reduction in Actinobacteriota and Proteobacteria and an increase in Firmicutes abundance.

### Children with SAM cannot effectively utilize inulin *ex vivo*

**Figure 2:**
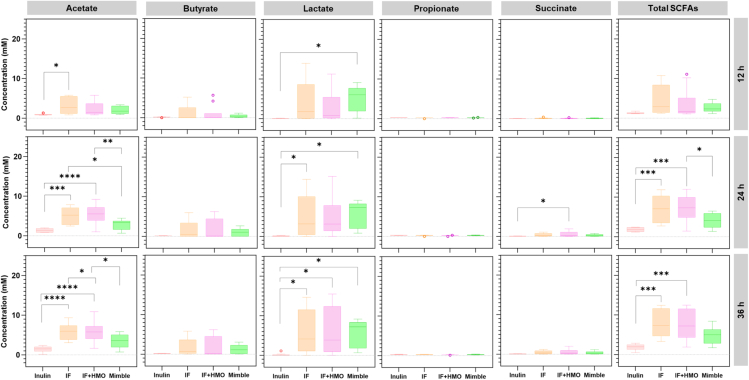
Production of microbial metabolites in the presence of different substrates The substrates tested were inulin, IF, IF + HMO, and chickpea-enriched feed (MIMBLE). Fatty acid concentration (mM) was determined using ^1^H NMR following 12, 24, and 36 h of *in vitro* fermentation. Statistically significant differences are indicated with ∗*p* value <0.05, ∗∗*p* value <0.01, ∗∗∗*p* value <0.001, ∗∗∗∗*p* value <0.0001. *n* = 10, data are expressed as mean ± SEM.

Microbial metabolites produced over 36 h of *in vitro* fermentation were quantified using ^1^H-nuclear magnetic resonance (NMR), and significant differences between substrates were observed (Figure 2). The total SCFA produced in the presence of inulin was significantly lower than from the other three substrates after 24 h of fermentation. The differences in total SCFAs were mainly driven by acetate and butyrate production. Acetate was highest for the two milk powders (IF and IF + HMO), followed by MIMBLE feed and then inulin. Butyrate levels were similar between IF, IF + HMO, and MIMBLE substrates, but lower for inulin. Propionate production was not significantly different (*p* > 0.05) across all substrates and time points, although it should be noted that propionate production was low in all the fermentations and only modest increases from baseline were seen with MIMBLE and IF substrates.

Metabolic intermediate products, such as lactate and succinate, were also different between substrates. Lactate was produced in response to MIMBLE, IF, and IF + HMO substrates at 12 h of fermentation and onward, while succinate production was delayed, peaking at 24 h of fermentation. Neither lactate nor succinate was produced in significant (*p* > 0.05) quantities in the presence of inulin. The absence of these metabolic intermediates would hinder the production of SCFA end products and could be the reason for lower butyrate and acetate concentrations for inulin compared to the other substrates tested in this study.^9^^,^^31^ The low production of intermediate metabolites is likely because of the limited breakdown of inulin by the fecal microbiota from children with SAM. Evidence for limited breakdown of inulin can be derived from the ^1^H NMR spectra of the fermentation media following 36 h of fermentation (Figure S2). Several peaks were observed only in the inulin-supplemented media, which can be assigned to inulin, including the peaks at 4.3 and 4.1 ppm, which arise from protons in the fructose ring of inulin.^32^ These inulin peaks remain invariant at all the time points, suggesting that the inoculum is unable to degrade and utilize inulin as a substrate throughout the fermentation experiments.

### Improvement in gut microbiota of children with SAM after 90 days of care

Generally, inulin is fermented by the gut microbiome of healthy children and adults,^33^^,^^34^^,^^35^^,^^36^^,^^37^^,^^38^^,^^39^^,^^40^ so it was hypothesized that the inability of children with SAM to utilize inulin *ex vivo* is because of the gut dysbiosis resulting from SAM complication and is not a characteristic of the population from which fecal samples were obtained. To test this hypothesis, in a follow-up trial, fecal samples were collected from children (*n* = 6) hospitalized with SAM on days 7 and 90 in the Mbale Regional Referral Hospital, Uganda, between July 2018 and August 2019. After 90 days of care, improvement in MUAC (from 11.9 cm on day 7 to 12.9 cm) and WHZ (from −2.2 on day 7 to −1.3) was observed (Table 2) with no cases of edema. Thus, at this time point, the cohort has persistence of mild acute malnutrition (rather than SAM). Day 90 samples, therefore, represent a partially anthropometrically recovered cohort.

**Table 2:** Demographics of children on day 7 and day 90 after hospitalization

Demographic	Day 7 (*n* = 6)	Day 90 (*n* = 6)
Age (years)	1.6 ± 0.3	1.8 ± 0.3
MUAC (cm)	11.9 ± 0.3 (10.6–13.1)	12.9 ± 0.4 (11.6–13.7)
Height (cm)	71.9 ± 3.8 (61.2–85.4)	73.9 ± 3.6 (62.0–86.9)
Weight (kg)	7.3 ± 0.9 (5.1–10.9)	8.4 ± 1 (5–12.2)
WHZ	−2.2 ± 0.4 (−3.53 to −1.27)	−1.3 ± 0.5 (−3.35 to 0.06)
Edema: ≥3 grade	2/6	0/6
Diarrhea	0/6	0/6
Vomiting	0/6	0/6

Data are presented as mean ± standard error of the mean.

Fecal samples collected on days 7 and 90 after hospitalization represent sick and partially anthropometrically recovered cohorts, respectively.

**Figure 3:**
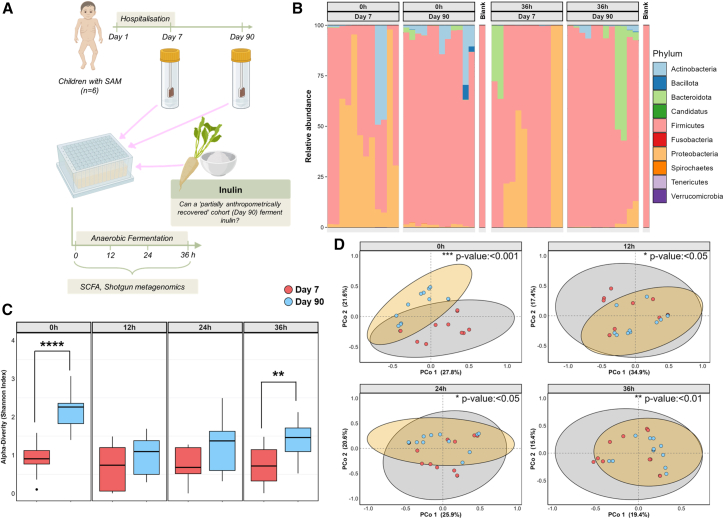
Changes in microbial composition during fermentation of inulin by day 7 and day 90 samples (A) Study design. Fecal samples were collected on days 7 and 90 after hospitalization from each participant (*n* = 6, analyzed in duplicate), representing sick and partially anthropometrically recovered cohorts, respectively. Microbial composition was determined by shotgun metagenomics using MetaPhlAn4 coupled with phyloseq and phylosmith pipelines. (B) The phylogeny profile shows the phylum-level abundances of bacterial taxa for individual participants at the start of the experiment (T = 0 h) and after 36 h of *in vitro* fermentation of inulin. (C and D) Changes in (C) alpha diversity (Shannon index) and (D) beta diversity (Bray-Curtis index) over time during *in vitro* fermentation of inulin. Statistically significant differences are indicated with ∗*p* value <0.05, ∗∗*p* value <0.01, ∗∗∗*p* value <0.001, ∗∗∗∗*p* value <0.0001; data are expressed as mean ± SEM.

**Figure 4:**
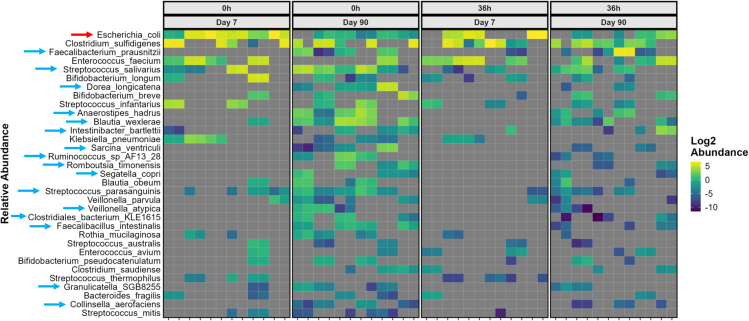
Species-level abundances during fermentation of inulin by day 7 and day 90 samples Species-level abundances of bacterial taxa for individual participants (*n* = 6, analyzed in duplicate) were determined by shotgun metagenomics using MetaPhlAn4 coupled with phylosmith and MaAsLin2 pipelines at the start of the experiment (T = 0 h) and after 36 h of *in vitro* fermentation of inulin. Species significantly higher in day 7 or day 90 cohorts at T = 0 h are marked with red and blue arrows, respectively. Supporting information on significance values is provided in Tables S2 and S3. Distribution at the genus level is provided in Figure S3.

The gut microbiome also improved with the treatment phase. Fecal samples collected on day 90 had significantly higher (almost double) alpha (Shannon index) diversity than day 7 samples (T = 0 h, Figure 3C). The observed difference in alpha diversity was underscored by a significant compositional difference between the groups, as samples clustered differently on a PCoA plot (T = 0 h, Permutational Multivariate Analysis of Variance (PERMANOVA), *p* value <0.001, Figure 3D). Initially, children had high Proteobacteria, particularly dominated by *Escherichia coli* and *Klebsiella pneumoniae* species, which on day 90 reduced to less than 1% phylum-level abundance (Figures 3B and 4). Contrarily, on day 90, children had high Firmicutes and some Bacteroidota, colonized by beneficial species like *F. prausnitzii, Streptococcus salivarius*, *Anaerostipes hadrus*, *Blautia wexlerae*, and *Faecalibacillus intestinalis*. This is consistent with previous observations reporting an association between anthropometric improvement and microbial community in malnourished children.^41^^,^^42^

### Treatment phase-dependent utilization of inulin

A miniaturized *in vitro* fermentation model was established to investigate the fermentability of inulin by the two groups. The model was inoculated with fecal samples collected from children (*n* = 6) either on day 7 or on day 90 after hospitalization (Figure 3A). With the progression of fermentation, a shift in microbial compositions was observed for both cohorts, as the clusters in PCoA plots deviated from the composition at T = 0 h and the composition remained significantly different between cohorts throughout the experiment (Figure 3D). Alpha diversity initially decreased for the day 90 cohort and became almost similar to that of the day 7 cohort but then recovered and was significantly higher at 36 h of fermentation (Figure 3C). In contrast, for the day 7 cohort, alpha diversity consistently remained low throughout the fermentation experiment. Fermentation also led to changes in phylum abundance, with an increase in Firmicutes observed for the day 7 and Bacteroidota for the day 90 samples after 36 h of fermentation (Figure 3B). At the genus level, *Enterococcus* dominated the day 7 group, while *Clostridium* increased in the day 90 group (Figure S3).

**Figure 5:**
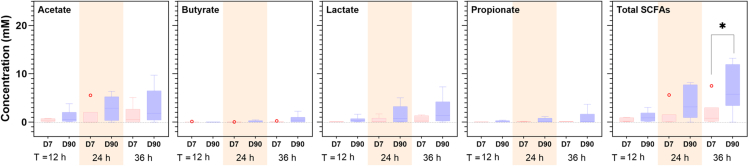
Production of microbial metabolites during fermentation of inulin by day 7 and day 90 samples Fecal samples were collected on day 7 and day 90 after hospitalization from each participant (*n* = 6, analyzed in duplicate), representing sick and partially anthropometrically recovered cohorts, respectively. Fatty acid concentration (mM) was determined using ^1^H NMR following 12, 24, and 36 h of *in vitro* fermentation. Statistically significant differences are indicated with ∗*p* value <0.05; data are expressed as mean ± SEM. D7, day 7; D90, day 90.

Alongside changes in the microbial community, the production of microbial metabolites was also assessed. The production of SCFAs (acetate, butyrate, and propionate) was consistently higher for the day 90 cohort across all time points compared to the day 7 cohort. However, significance was only achieved for the total SCFA production at 36 h of fermentation (Figure 5). Furthermore, there was an increasing trend in the production of acetate, lactate, propionate, and total SCFAs with fermentation time for both day 7 and day 90 groups, but statistically significant increases were only reached for acetate and total SCFAs in the day 90 group after 24 h of fermentation, compared to baseline (Figure S4). Collectively, higher diversity and total SCFA production during fermentation suggest that the day 90 cohort can effectively utilize inulin.

### Differences in carbohydrate-active enzymes

**Figure 6:**
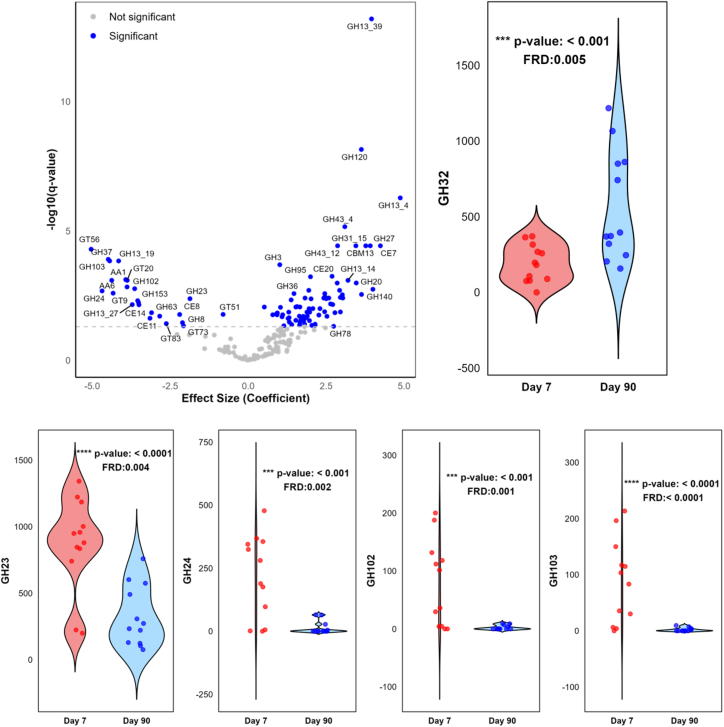
CAZyme families in day 7 and day 90 samples Fecal samples were collected on day 7 and day 90 after hospitalization from each participant (*n* = 6, analyzed in duplicate), representing sick and partially anthropometrically recovered cohorts, respectively. CAZyme families were determined by shotgun metagenomics using dbCAN3 coupled with the MaAsLin2 pipeline at the start (T = 0) of *in vitro* fermentation of inulin. The volcano plot shows the CAZyme families present in the cohorts, with statistically different (*p* value <0.05) families marked in blue circles above the horizontal dotted line, signifying *q* < 0.05 after mixed effect model adjustment for covariates. Supporting information is provided in Table S4. Violin plots show abundances of selected CAZyme families. Statistically significant differences between specific families shown in the violin plot are indicated with ∗∗∗*p* value <0.001, ∗∗∗∗*p* value <0.0001; data are expressed as mean ± SEM.

The differences in microbial community between the day 7 and day 90 cohorts could lead to differences in the presence or abundance of carbohydrate-active enzymes (CAZymes), affecting their ability to metabolize carbohydrates. At T = 0 h, 110 CAZyme families exhibited significantly different abundances between the two cohorts, with most belonging to glycoside hydrolase (GH, 69 families) and glycosyl transferase (16 families) classes (Figure 6; Table S4). The day 90 cohort had notably higher levels of enzyme families such as GH13, GH27, GH31, GH32, GH43, and GH120, which are involved in carbohydrate metabolism, suggesting they can utilize a variety of carbohydrates as substrates. A high abundance of the GH32 family, which includes β-fructosidase responsible for inulin degradation,^43^ indicates that the day 90 cohort had the potential to effectively break down inulin and utilize it as a substrate during *in vitro* fermentation. Conversely, the day 7 cohort was enriched in families such as GH23, GH24, GH102, and GH103, among others, which are involved in peptidoglycan hydrolysis, implying differences in the functional potential of the cohorts. However, as fermentation continued, there were no significant differences (*p* > 0.05) in CAZyme families between the cohorts at later time points (Figure S5).

## Discussion

The gut microbiome has recently emerged as an important target in the short- and long-term nutritional treatment of SAM. A series of studies have indicated that SAM is associated with gut microbiome immaturity,^15^^,^^16^^,^^18^^,^^44^ leading to a reduced production of beneficial microbial metabolites, such as SCFAs, crucial to healthy gut function. Reduced fecal SCFAs have been associated with increased mortality in children with SAM.^25^^,^^45^ Moreover, the gut microbiome could be further damaged by the extensive use of antibiotics as part of the initial SAM treatment regimen. Dietary interventions including ingredients that specifically target the gut microbiome, such as legume-based sources, have demonstrated that the additional stunting of diversity in the first week of treatment is ameliorated by legume-supplemental feeds.^25^

Here, we adapted *in vitro* colon models to mimic the gut microbiome of SAM patients and showed selective utilization of carbohydrate substrates. The basal media used in models was a minimal medium with only key vitamins and minerals for microbial growth, but no additional carbon sources, reflecting the restricted diet of SAM patients. To establish the gut microbiota of the SAM patients *in vitro*, fecal samples collected from children with SAM were used as inoculum. The fecal samples were selected from patients who had been in clinical treatment for SAM for 7 days following a standard WHO treatment pathway and, therefore, reflected the combined impact of SAM, standard WHO milk-based nutritional feeds, antibiotic treatment, and other co-morbidities like Tuberculosis and malaria on the gut microbiota of SAM patients.^25^ Fecal samples from these children had high levels of Proteobacteria and very low levels of Bacteroidetes (<5% in all samples), consistent with previous studies of childhood malnutrition.^15^^,^^19^^,^^46^ The high abundance of Proteobacteria was also observed in the larger cohort, from which these samples were obtained,^25^ where Proteobacteria levels were found to peak at day 7 following hospital admission, the point at which the samples in our study were taken. Strikingly, the gut microbiome of these children was enriched with pathobionts, namely, *Escherichia coli*, *Enterococcus faecium*, and *K. pneumoniae*, which could contribute to severe infections in children with malnutrition.^47^^,^^48^^,^^49^

Using our *in vitro* model system, we illustrated that inulin is not completely fermented by the gut microbiota of children with SAM. Recent animal model studies and an intervention study in children with SAM found similar results, with inulin failing to increase fecal SCFAs or increase weight gain.^25^^,^^50^ However, several studies have demonstrated that fecal samples from healthy pre- and post-weaning infants were capable of degrading inulin to produce SCFAs within 12–24 h, which is well within the time course of the present study.^34^^,^^35^^,^^36^^,^^37^ This suggests that the immaturity and high pathobiont burden of the gut microbiome of children with SAM could potentially hinder the utilization of inulin.

Furthermore, studies investigating the *in vitro* fermentation of inulin by fecal samples from infants have also shown a clear age-dependent effect, where the gut microbiome of very young infants cannot ferment inulin, while older infants can partially ferment lower-molecular-weight inulin.^34^^,^^35^ In order to confirm that the poor utilization of inulin results from microbiome immaturity during SAM rather than the age of the population, we also investigated the fermentation of inulin by the fecal microbiome from children after partial anthropometric recovery (90 days after hospitalization with SAM). We confirmed that the gut microbes of the partially anthropometrically recovered cohort could better utilize inulin during fermentation and produce elevated levels of SCFAs. The utilization of inulin by this cohort may be a result of improved gut microbiome diversity (characterized by increased alpha diversities), with high levels of Firmicutes and very low levels of Proteobacteria. This favorable shift in gut microbial composition was reflected by the presence of distinct species from genera such as *Faecalibacterium*, *Streptococcus*, *Anaerostipes*, and *Blautia*, which are commonly found in healthy children.^51^^,^^52^ *Bifidobacterium* species such as *Bifidobacterium longum*, *Bifidobacterium breve*, *Bifidobacterium pseudocatenulatum*, and *B. fragilis*, involved in inulin fermentation, were also present, although their abundance did not increase during *in vitro* fermentation. In adults, species of the genus *Bifidobacteria* and *Bacteroides* have been reported to be involved in inulin fermentation.^53^ The improved gut microbial composition during SAM recovery also enhanced the functional potential of the gut, indicated by high levels of GH32 CAZyme family, which is a key player in inulin metabolism.^43^

In contrast, the MIMBLE feed, which is enriched with legumes, and milk powders (with and without HMO) were found to be fermentable during the initial treatment phase of SAM (day 7). Animal studies have demonstrated that the HMO 2′-FL contributes to increased SCFA output and supports weight gain in a model of SAM.^50^ In the present study, the fermentability of the two milk formulas tested was very similar, and the SCFA output of both was high. In contrast to HMOs, legumes are cheap, widely produced, and locally available in countries most affected by malnutrition, therefore making an excellent candidate for addition to supplementary feeds for use in the treatment of SAM. In the present study, we tested the MIMBLE feed, containing 10% chickpea flour, which was found to be fermented by the gut microbiota of children with SAM in an *in vitro* model, yielding a range of SCFAs. The chickpea-enriched feed also resulted in a greater reduction in Proteobacteria abundance during fermentation than the other substrates tested. Several recent studies have identified that feeds containing chickpea flour have the potential to act as a food source for the gut microbiota of children with SAM, due to the diverse range of polysaccharides, including resistant starch, pectin, and arabinoxylans present in chickpea. These polysaccharides can be used to modulate the gut microbiome and promote the growth of beneficial bacteria in the immature gut microbiota of SAM patients.^21^^,^^42^^,^^54^ These results indicate the potential for the use of locally sourced plant-based foods, such as chickpeas, for use in recovery foods, which can support SCFA production in the colon as effectively as HMOs.^23^^,^^42^^,^^55^

In this study, we established two *in vitro* models of the gut microbiome of children with SAM to test the fermentability of four different carbohydrate substrates. Children with SAM had low diversity with high Proteobacteria and low Bacteroidetes abundance. We demonstrated that, while two milk powders and a legume-based feed were fermentable and produced SCFAs in the model, inulin was not fermented to a significant degree. This inability to ferment inulin was confirmed to be because of limited microbial diversity in children with SAM, as the same children, after receiving standard treatment for 90 days, could utilize inulin. These results demonstrate that assumptions cannot be made regarding the fermentability of carbohydrates by the gut microbiota of children with SAM and that results obtained in healthy childhood cohorts are not translatable to children with SAM. This is, therefore, an urgent need for future studies screening the fermentability of carbohydrates by the gut microbiota of children with SAM, targeting different stages of the recovery period. The results presented in this paper provide insights useful for the development of therapeutic and complementary feeds for use during treatment and recovery from SAM.

### Limitations of the study

A limitation of this study is that the food samples were not matched for their nutrient composition. Although carbohydrates were the predominant macronutrient and likely the main drivers of fermentation, the low levels of protein and fat present may still have exerted minor influences on microbial metabolism. Additionally, the paired assessment of *in vitro* inulin utilization at days 7 and 90 post-hospitalization was limited to six children due to high dropout rates.

## Resource availability

### Lead contact

Requests for further information and resources should be directed to and will be fulfilled by the lead contact, Frederick J. Warren (fred.warren@quadram.ac.uk).

### Materials availability

This study did not generate new unique reagents.

### Data and code availability

• The raw sequencing data used in this manuscript can be accessed through the NCBI SRA project number PRJNA1080518 (https://www.ncbi.nlm.nih.gov/bioproject/PRJNA1080518).
• This paper does not report original code.
• Any additional information required to reanalyze the data reported in this article is available from the lead contact upon request.

## Acknowledgments

We gratefully acknowledge the technical assistance of David Baker with library preparation and Alise Ponsero with the dbCAN pipeline setup. We thank all the participants and trial staff who participated in the MIMBLE trials. The authors gratefully acknowledge the support of the 10.13039/501100000268Biotechnology and Biological Sciences Research Council (BBSRC); this research was funded by the 10.13039/501100000268BBSRC Institute Strategic Programme Food Microbiome and Health BB/X011054/1 and its constituent projects BBS/E/QU/230001A and BBS/E/QU/230001B.

## Author contributions

F.J.W. conceptualized the study; A.B., J.A.-J., K.C., and S.H. performed experimental work, data collection, and formal analysis; P.T.-R. contributed to bioinformatic analysis; P.O.-O., N.C., K.W., K.M., and G.F. contributed to MIMBLE 1 and 2 trials from which fecal samples were obtained; A.B., J.A.-J., and F.J.W. contributed to writing – original draft. All authors have read and contributed to the manuscript.

## Declaration of interests

The authors declare no competing interests.

## STAR★Methods

### Key resources table

**Table:** 

REAGENT or RESOURCE	SOURCE	IDENTIFIER
**Biological samples**		

Faecal samples from children with Severe Acute Malnutrition (SAM)	Calder et al.^25^ and Walsh et al.^26^	N/A

**Chemicals, peptides, and recombinant proteins**		

Inulin from chicory	Sigma-Aldrich	Cat#I2255
D_2_O	Merck Life Sciences	Cat#151882

**Critical commercial assays**		

FastDNA® Spin Kit for Soil	MP Biomedical	Cat#116560200
QuantiFluor® dsDNA System	Promega	Cat#E2670
Kap2G Robust PCR kit	Sigma-Aldrich	Cat#KK5005
Nextera XT Index Kit v2	Illumina	Cat#FC-131-2001

**Deposited data**		

Raw metagenomic data	This paper	NCBI SRA project number PRJNA1080518 (https://www.ncbi.nlm.nih.gov/bioproject/PRJNA1080518)

**Software and algorithms**		

QIIME2	Bolyen et al.^56^	Version 2020.11
DADA2	Callahan et al.^57^	N/A
Silva Database	Quast et al.^58^	https://www.arb-silva.de/
MetaPhlAn4	Beghini et al.^59^	https://huttenhower.sph.harvard.edu/biobakery_workflows/
dbCAN3	Zheng et al.^60^	https://bcb.unl.edu/dbCAN2/
ChocoPhlAn	Blanco-Míguez et al.^61^	N/A
Megahit	Li et al.^62^	Version 1.2.9
Prokka	Seemann et al.^63^	Version 1.14.6
PhyloSeq R packages	McMurdie et al.^64^	https://www.bioconductor.org/packages/release/bioc/html/phyloseq.html
PhyloSmith R package	Smith et al.^65^	https://schuyler-smith.github.io/phylosmith/index.html
MaAsLin2 R package	Mallick et al.^66^	https://huttenhower.sph.harvard.edu/maaslin2

**Other**		

NMR tubes (Wilmad® economy, 5 mm)	Merck Life Sciences	Cat#Z565229
Similac Pro-Advance with human milk-like oligosaccharide	Amazon	N/A
SMA Pro 3 Toddler milk	Boots Pharmacists	N/A
Chickpea-based feed (MIMBLE)	Walsh et al.^67^	N/A

### Experimental model and study participant details

#### Inoculum collection and preparation

Faecal samples used in the screening study were obtained from the modifying intestinal integrity and microbiome in malnutrition with legume-based feeds 1 (MIMBLE 1) trial. MIMBLE 1 was a single-centre (Mbale Regional Referral Hospital) proof-of-principle, randomised comparator trial evaluating the safety and feasibility of three feeding strategies.^25^ The protocol was approved by the ethics committees of Imperial College London (15IC3006) and Mbale Regional Referral Hospital (UG-IRC-012). Following parental written consent, children aged 6 months to 5 years were enrolled on day 1 post-admission and followed for 28 days. The trial was conducted to the standards of ICH GCP. Children hospitalised with SAM were screened for eligibility (one or more of mid-upper arm circumference (MUAC) <11.5 cm, weight-for-height Z-score (WHZ) < -3 or Kwashiorkor) and randomised to either standard milk-feed F75 (*n* = 18), inulin-supplemented standard feeds (*n* = 20) or cowpea-supplemented standard feed (*n* = 20). Faecal samples were obtained on day 7 following admission to the hospital from trial participants (*n =* 10) receiving the standard WHO F75/F100 feeding regime and other supportive therapies, including antibiotics. Stools from children on inulin or legume-based feeds were not recruited to this study. Faecal samples were frozen and stored at -80°C prior to use.

For the inulin study, faecal samples were obtained from modifying intestinal integrity and microbiome in malnutrition with legume-based feed 2 (MIMBLE 2) trial. MIMBLE 2 was a single-centre (Mbale Regional Referral Hospital) proof-of-principle, randomised controlled trial evaluating the safety and efficacy of lactose-free chickpea formula compared to milk-based feeds.^26^ The protocol was approved by the ethics committees of Imperial College London (17IC4146) and Mbale Regional Referral Hospital (019/2018). Children aged 6 months to 5 years, hospitalised with SAM, were screened for inclusion criteria and were enrolled in the trial following parental written consent. Children were assigned to either the standard WHO feed (*n* = 80) or the chickpea-rich feed (*n* = 80) and were reviewed on day 7, 28 and 90 post-hospitalisation. Faecal samples received on day 7 and day 90 following admission to the hospital, from trial participants (*n =* 6) receiving the standard WHO F75/F100 feeding regime, were used in this study. Faecal samples from children on chickpea-based feeds were not included. Faecal samples were frozen and stored at -80°C before use.

To prepare the inoculum, the samples were thawed at room-temperature, diluted 1:10 with pre-warmed, anaerobic, sterile phosphate buffer saline (0.1M, pH 7.4) and mixed well either in a double meshed stomacher bag (500 mL, Seward, Worthing, UK; homogenized at 200 rpm for 2 cycles, 60 sec each) or pipetting multiple times, depending on sample size.

### Method details

#### Substrates

Chicory inulin (catalogue no. I2255) was purchased from Sigma-Aldrich (Gillingham, UK). Similac Pro-Advance with human milk-like oligosaccharide (IF+HMO) was purchased from Amazon (UK), and SMA Pro 3 Toddler milk (IF) was purchased from Boots Pharmacists (UK). Chickpea-based feed (MIMBLE) was prepared by Campden BRI as described elsewhere.^67^ Nutrient composition (Table S1) was approximated using Nutritics software (Ireland) or using manufacturer-provided information.

#### Batch fermentation

Each substrate (0.500 ± 0.005 g, dry weight) was weighed into sterilised fermentation bottles (100 mL) prior to the start of the experiment. Fermentation experiments were performed with media adapted from Warren and colleagues (2018).^68^ In brief, fermentation vessels (100 mL) each contained an aliquot of faecal inoculum (3 mL), sterilised growth medium (82 mL), and substrate. The growth medium was prepared by mixing basal solution (76 mL), vitamin phosphate and sodium carbonate solution (5 mL), and reducing agent (1 mL). The composition of the solutions used in the preparation of the growth medium is described elsewhere.^69^ A single stock (7 L) of growth medium was used for all vessels prepared for this experiment. Vessel fermentations were pH controlled and maintained at pH 6.8 to 7.2 using 1N NaOH and 1N HCl regulated by a Fermac 260 (Electrolab Biotech, Tewkesbury, UK). A circulating water jacket maintains vessel temperature at 37°C. A magnetic stirrer was used to keep the mixture homogenous, and the vessels were continuously sparged with nitrogen (99% purity), maintaining anaerobic conditions. Media with no inoculum was used as a blank, whereas inulin, IF, IF+HMO and chickpea feed (MIMBLE) with inoculum were the experimental conditions evaluated. Samples were collected at 0 (∼5 min post inoculation), 6, 12, 24, 36 and 48 h after inoculation.

A miniaturised *in vitro* fermentation model was set up in a 2 mL-deep 96-well plate, using the medium described in the screening study. Briefly, inulin (5.88 mg/mL) was added to sterilised growth medium as described above and mixed using a magnetic stirrer for 2 h at room temperature, followed by purging with CO_2_ for 30 min. From the resulting mix, 1.64 mL was transferred into each well of the deep 96-well plate in an anaerobic cabinet (5% CO_2_, 10% H_2_ and 85% N_2_; Baker Ruskinn Concept 1000), resulting in ∼10 mg of inulin per well, and incubated to equilibrate overnight. The following day, faecal inoculum was prepared, and 60 μL was added to each well. The plates were sealed with a gas-permeable adhesive seal (catalogue no. E2796-3015, Starlab, Hamburg, Germany) and incubated anaerobically at 37°C for 0 (∼2 min post inoculation), 12, 24 and 36 h without mixing. Different plates were set up for each time point. Fermentation was carried out in duplicate for each sample.

At fixed time points, samples were taken from the fermentation mix and centrifuged (13,000 x *g*, 5 min, 4°C). The supernatants were collected in fresh tubes and stored at -20°C for ^1^H NMR metabolomic analysis, while the bacterial pellets were stored at -80°C until DNA extraction.

#### ^1^H NMR metabolomic profiling

The samples containing the supernatant from the fermentation media were centrifuged (13,000 x *g*, 3 min) and 400 μL of the supernatant was pipetted directly into NMR tubes (Wilmad® economy, 5 mm), followed by the addition of 200 μL of NMR buffer prepared in D_2_O with following concentrations of reagents: 21.7 mM NaH_2_PO_4_, 82.7 mM K_2_HPO_4_, 8.6 mM NaN_3_, 1.0 mM 3-(trimethylsilyl)-propionate-d_4_ (TMSP).

For the screening study, spectra were collected on a Bruker NEO 600 MHz spectrometer equipped with a cryoprobe. All experiments were acquired at room temperature, using Bruker’s ‘noesygppr1d’ pulse sequence, with a minimum of 64 scans. A 90° pulse length of 11.09 μs was set for all samples with a mixing time of 0.01 s, acquisition time of 2.62 s, relaxation delay of 4 s, featuring selective pre-saturation (1.0 ms) on the residual H_2_O peak frequency during relaxation delay and mixing time for effective solvent suppression.

For the inulin study, spectra were acquired on a Bruker Avance III NMR 500 MHz spectrometer equipped with a BBO z-gradient probe. The ^1^H NMR spectra were recorded at room temperature using Bruker’s WATERGATE pulse sequence. All other parameters were: a minimum of 128 scans, spectral width of 14.994 ppm, centred on at 4.702 ppm, acquisition time of 4.37 s, and relaxation delay of 4 s.

All spectra were automatically phased, and a baseline correction was applied. Spectra were referenced using the TMSP peak (δ = 0.0 ppm) for internal chemical shift. The metabolites were quantified using the NMR Suite Profiler v10.0 (Chenomx®, Edmonton, Canada). Total SCFA concentration was calculated by adding acetate, butyrate and propionate concentrations.

#### DNA extraction

The frozen bacterial pellets were resuspended in 400 μL of nuclease-free water (Sigma-Aldrich, Gillingham, UK), and DNA extraction was performed using the FastDNA® Spin Kit for Soil (MP Biomedical, Solon, USA), according to the manufacturer’s instructions. Cell lysis was carried out using the FastPrep bead-beating instrument (FastPrep24, MP Biomedical, Solon, USA) at a speed of 6.0 m/s (3 runs of 40 sec each, 5 min rest on ice in between runs). DNA concentration was determined using the QuantiFluor® dsDNA System (Catalogue No. E2670, Promega, UK) and quantified on a FLUOstar Optima plate reader (BMG Labtech, Aylesbury, UK). Extracted genomic DNA was normalised to 5 ng/μL with elution buffer (10 mM Tris-HCl).

#### Library preparation and sequencing

For the screening study (16S rRNA sequencing), a PCR master mix was made up using 4 μL KAPA 2G buffer, 0.4 μL dNTP’s, 0.08 μL Polymerase, 0.4 μL 10 μM forward tailed specific primer, 0.4 μL 10 μM reverse tailed specific primer and 12.72 μL PCR grade water (contained in the Kap2G Robust PCR kit Sigma Catalogue No. KK5005) per sample and 18 μL added to each well to be used in a 96-well plate followed by 2 μL of DNA and mixed. Specific PCR conditions were 95^0^C for 5 min, 30 cycles of 95^0^C for 30 s, 55^0^C for 30 s and 72^0^C for 30 s, followed by a final 72^0^C for 5 min. Following PCR, a 0.7X SPRI clean-up was performed using KAPA Pure Beads (Roche Catalogue No. 07983298001), eluting the DNA in 20 μL of water. A second PCR master mix was made up using 4 μL KAPA 2G buffer, 0.4 μL dNTP’s, 0.08 μL Polymerase, and 6.52 μL PCR grade water per sample and 11 μL added to each well to be used in a 96-well plate. 2 μL of each P7 and P5 of Nextera XT Index Kit v2 index primers (Illumina Catalogue No. FC-131-2001 to 2004) were added to each well. Finally, the 5 μL of the clean, specific PCR was added and mixed. The second PCR was run using 95^0^C for 5 min, 10 cycles of 95^0^C for 30 s, 55^0^C for 30 s and 72^0^C for 30 s, followed by a final 72^0^C for 5 min. Final libraries were quantified by Qubit and equimolar pooled together. A single 0.7X SPRI clean-up was performed on the pool. A final Qubit and sizing on High Sensitivity D1000 Screen Tape (Agilent Catalogue No. 5067-5579) using the Agilent Tapestation 4200 was done to calculate the final library pool molarity. The pool was run at a final concentration of 10 pM on an Illumina MiSeq instrument using MiSeq® Reagent Kit v3 (600 cycle) (Illumina Catalogue FC-102-3003) following the Illumina recommended denaturation and loading recommendations, which included a 20% PhiX spike in (PhiX Control v3 Illumina Catalogue FC-110-3001). The raw data was analysed locally on the MiSeq using MiSeq reporter.

For inulin study (shotgun metagenomics), 0.5 μL of tagmentation buffer (TB1) was mixed with 0.5 μL bead-linked transposomes (BLT) (Illumina Catalogue No. 20018704) and 4 μL PCR grade water in a master mix and 5 μL added to a 96-well plate. 2 μL of normalised DNA (10 ng total) was pipette mixed with the 5 μL of the tagmentation mix and heated to 55^0^C for 15 min in a PCR block. A PCR master mix was made up using 10 μL KAPA 2G Fast Hot Start Ready Mix (Merck Catalogue No. KK5601) and 2 μL PCR-grade water per sample. 12 μL of this master mix was added to each well to be used in a 96-well plate. 1 μL of 10 μM 8bp Unique Dual Indexes was added to each well. Finally, the 7 μL of tagmentation mix was added and mixed. The PCR was run with 72^0^C for 3 min, 95^0^C for 1 min, 14 cycles of 95^0^C for 10 s, 55^0^C for 20 s and 72^0^C for 3 min. The libraries were quantified using the Promega QuantiFluor® dsDNA System (Catalogue No. E2670) and run on a GloMax® Discover Microplate Reader. Libraries were pooled following quantification in equal quantities. The final pool was double-SPRI size selected between 0.5 and 0.7X bead volumes using sample purification beads (Illumina® DNA Prep, (M) tagmentation (96 Samples, IPB), 20060059). The final pool was quantified on a Qubit 3.0 instrument and run on a D5000 ScreenTape (Agilent Catalogue No. 5067-5579) using the Agilent Tapestation 4200 to calculate the final library pool molarity. The pool was sent to Novogene Company Ltd (Cambridge, UK) for shotgun metagenomic sequencing using Illumina NovaSeq X Plus (single lane, 10B flow cell) to generate 150 bp paired-end libraries with a sequencing depth of ∼23 million reads per sample.

#### Bioinformatics analysis

The demultiplexed FASTQ files were used for the bioinformatics analysis. 16S sequencing dataset (screening study) was processed for taxonomic analysis using QIIME2 (version 2020.11) with default parameters unless otherwise stated.^56^ DADA2 was used for paired-end joining, quality filtering, denoising and calling amplicon sequence variants (ASV’s) using the QIIME DADA2 denoise-paired method.^57^ The first 13 bp and the final 50 bp were trimmed before merging due to lower quality scores. A total of 855 ASV’s were identified across all the samples. Samples with fewer than 1000 reads were excluded from subsequent analysis, and the data were rarefied to 17,599 reads. The ASV’s were aligned and used to calculate a phylogenetic tree using the QIIME function phylogeny align to tree.^70^^,^^71^ Taxonomic assignment of the ASV’s was performed using the QIIME naïve Bayesian classifier Scikit-learn using the Silva 99% OTU database.^58^^,^^72^^,^^73^

Shotgun metagenomics data (inulin study) was processed using bioBakery 3 tools,^59^ including KneadData—for quality control and Metagenomic Phylogenetic Analysis 4 (MetaPhlAn4)—for taxonomic profiling and a separate tool, dbCAN3—to annotate carbohydrate-active enzymes (CAZymes).^60^ Firstly, the raw sequences were processed using KneadData to remove adaptor sequences, trim bases with low quality and short reads and remove possible contaminations from host DNA or bacterial rRNA. These high-quality, trimmed and non-human reads were then subsequently used in MetaPhlAn4 and dbCAN3 analyses. MetaPhlAn4 was used to examine what microbes are present and at what abundances by mapping the reads to the ChocoPhlAn database (vJan21_CHOCOPhlAnSGB_202103) of unique clade-specific marker genes.^61^ Processed reads after KneadData were assembled into contigs using Megahit (version 1.2.9)^62^ and gene prediction was done using Prokka (version 1.14.6)^63^ before running dbCAN3. dbCAN3 (version 4.1.4) was used to calculate the abundance of CAZyme families by mapping predicted CAZyme and CAZyme gene clusters (CGCs) from HMMER, HMMdb and DIAMOND against the Cazy database. Data visualisation was carried out using the PhyloSeq^64^ and PhyloSmith^65^ R packages.

### Quantification and statistical analysis

#### Statistical analysis

Metabolite data were analysed using GraphPad Prism (version 10.1.0, GraphPad Software, USA). The significant differences in SCFAs concentrations between substrates or within a substrate across the fermentation time were examined using a one-way ANOVA test with post-hoc Tukey’s HSD, at a p-value < 0.05. The significant differences in SCFAs concentrations between day 7 and day 90 samples at a specific fermentation time point were examined using a two-tailed paired t-test, at a p-value < 0.05.

The significant differences in alpha (Shannon index) and beta (Bray–Curtis distance matrix) diversity at a specific fermentation time point were tested using Kruskal-wallis with Wilcox test (pairwise) and PERMANOVA, respectively, in R using the vegan^74^ package with the adonis2 function (version 4.4.3, R Studio, USA).

Multivariate association analysis (MaAsLin2)^66^ was used to determine significant differences in the abundance of (i) species and (ii) CAZyme families between day 7 and day 90 samples. Parameters for MaAsLin2 were: linear mixed-effects model (LM), TSS normalisation, log transformation, and a minimum prevalence of 0.2 whilst controlling for faeces collection day as a fixed effect. Corrected significant p-values (< 0.05) were estimated using the Benjamini-Hochberg (BH) correction method.
