# Supplementary material for: Gut microbial diversity impacts carbohydrate fermentation by children with severe acute malnutrition
Source: iScience. 2026 Jan 7;29(2):114640. doi: 10.1016/j.isci.2026.114640 (PMC12857371; doi:10.1016/j.isci.2026.114640)
Supplement: Document S1. Figures S1–S5 and Tables S1–S4 [file mmc1.pdf]

iScience, Volume *29*

Supplemental information

Gut microbial diversity impacts carbohydrate fermentation by children with severe acute malnutrition

Akshay Bisht, Jennifer Ahn-Jarvis, Kendall Corbin, Suzanne Harris, Perla Troncoso-Rey, Peter Olupot-Olupot, Nuala Calder, Kevin Walsh, Kathryn Maitland, Gary Frost, and Frederick J. Warren

Supplementary Information

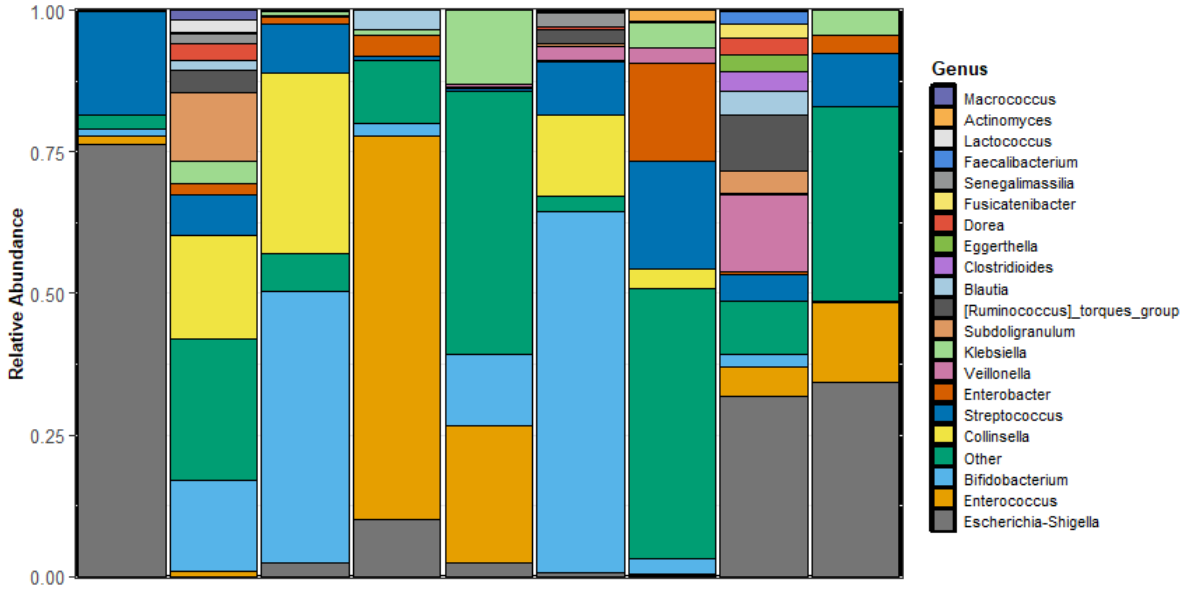

Figure S1. Phylogeny profile showing the genus-level abundances of bacterial taxa for individual participants. Abundances were determined by 16S sequencing at the start of the experiment (T = 0 h), prior to *in vitro* fermentation. The 20 most abundant genera are shown.

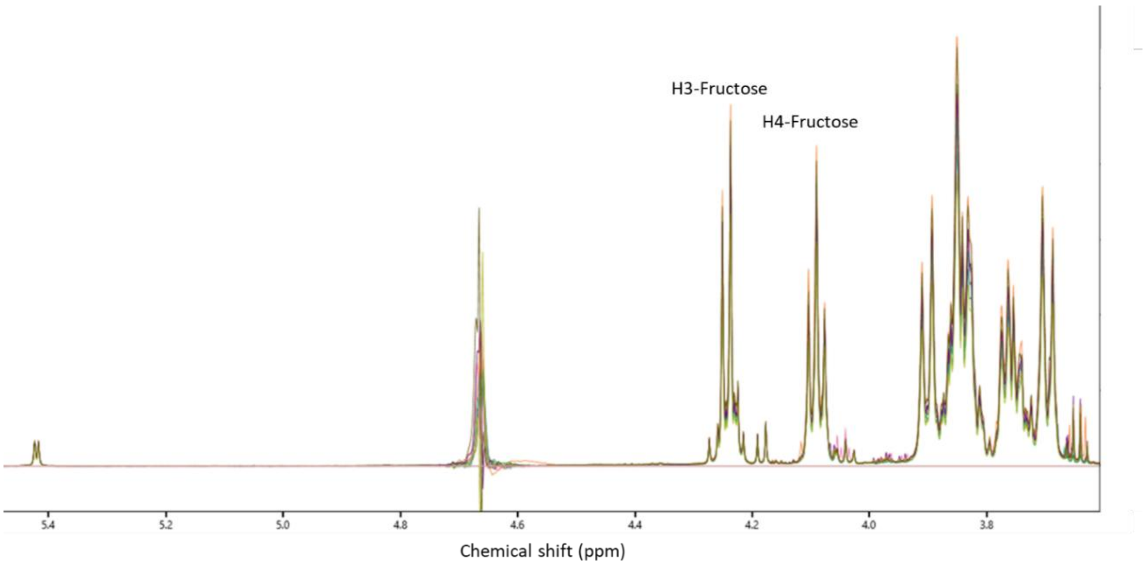

Figure S2. ^{1}H NMR spectra for fermentation media sampled from the inulin substrate fermentation vessels following 36 h of fermentation. Peak assignments from Caleffi et al.[1] for the H3 and H4 protons in the fructose ring.

[1] Caleffi, E. R. *et al.* Isolation and prebiotic activity of inulin-type fructan extracted from Pfaffia glomerata (Spreng) Pedersen roots. *International Journal of Biological Macromolecules* **80**, 392-399 (2015).

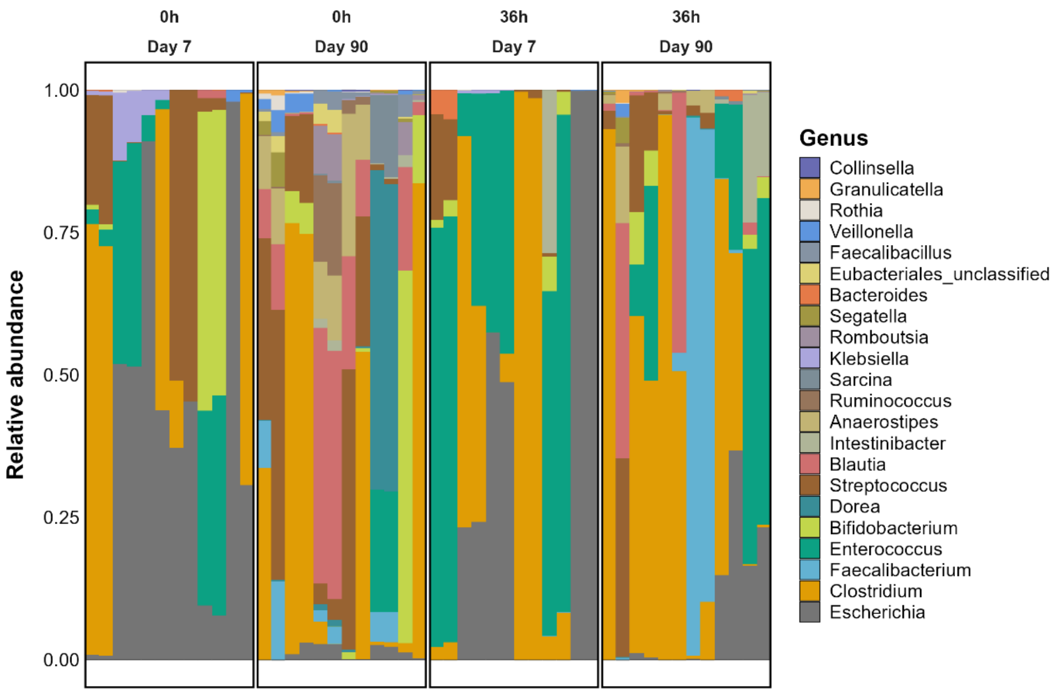

Figure S3. Genus-level abundances of bacterial taxa during fermentation of inulin by day 7 and day 90 samples. Abundances were determined by shotgun metagenomics using MetaPhlAn4 coupled with the phylosmith pipeline at the start of the experiment (T = 0 h) and after 36 h of *in vitro* fermentation of inulin.

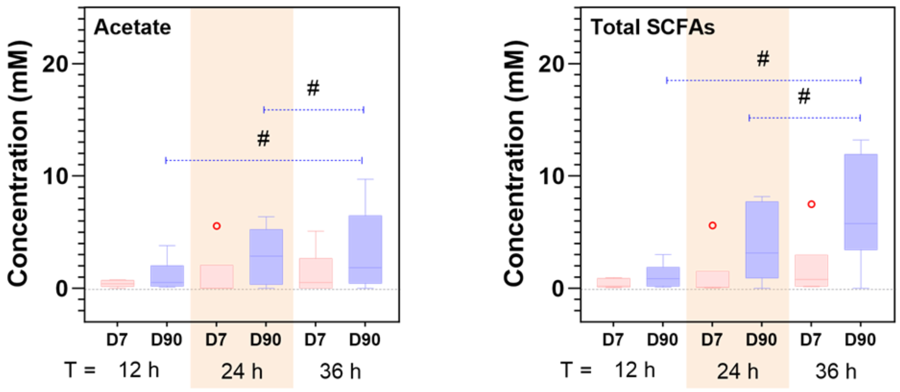

Figure S4. Acetate and total SCFAs production during fermentation of inulin in the presence of day 7 and day 90 faecal inoculum. Fatty acid concentration (mM) was determined using ^{1}H NMR following 12, 24 and 36 h of *in vitro* fermentation. Compared the effect of fermentation times for day 7 (D7) and day 90 (D90) samples using One-way ANOVA with a post-hoc Tukey's test. Statistically significant differences are indicated with # p-value < 0.05.

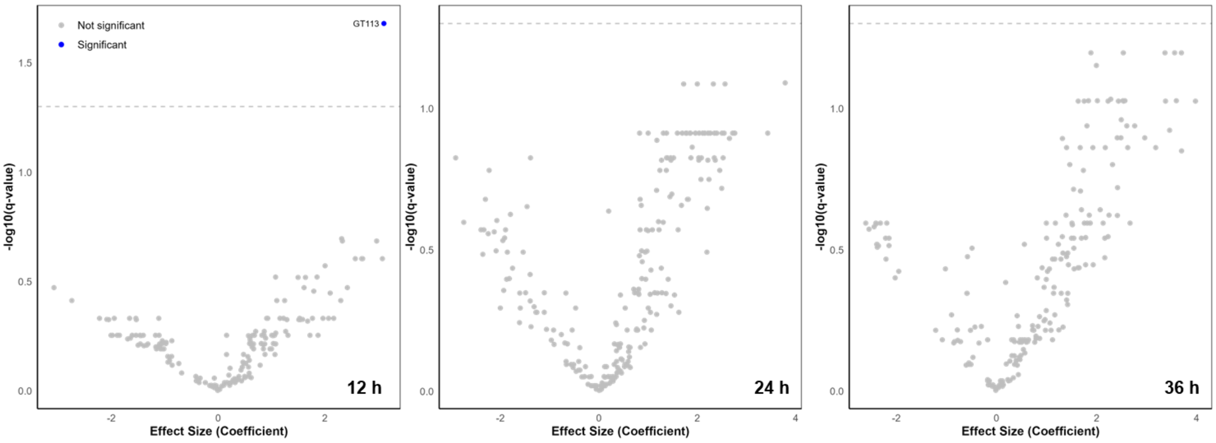

Figure S5. Comparison of CAZyme families during fermentation of inulin in the presence of day 7 and day 90 faecal inoculum. From each participant (*n* = 6), faecal samples were collected on day 7 and day 90 after hospitalisation, representing sick and partially anthropometrically recovered cohorts, respectively. CAZyme families were determined by shotgun metagenomics using dbCAN3 coupled with the MaAsLin2 pipeline at 12 h, 24 h and 36 h of *in vitro* fermentation of inulin.

Table S1. Nutritional composition of the substrates.

Nutrition Information	IF + HMO (Similac Pro-Advance†)	IF (SMA Pro 3 Toddler Milk‡)	MIMBLE‡	Inulin
Nutrient composition				
Energy (kcal)	6.1	4.64	2.02	2.1
Fat (g)	0.34	0.24	0.12	-
Linoleic acid (mg)	60.98	38.18	25.0	-
Total carbohydrates (g)	0.64	0.50	0.18	0.98
Dietary fiber (g)	0.0	0.0	0.02	0.90
Protein (g)	0.12	0.10	0.04	-
***Vitamins*‡**				-
Vitamin A (µg)	5.48	4.34	0.40	-
Vitamin D (µg)	0.12	0.08	0.00	-
Vitamin E (mg)	0.06	0.06	0.02	-
Vitamin K (µg)	0.48	0.48	0.14	-
Thiamin (µg)	6.10	7.24	0.70	-
Riboflavin (µg)	9.76	18.12	0.04	-
Vitamin B6 (µg)	3.84	4.34	0.90	-
Vitamin B12 (µg)	0.02	0.02	0.00	-
Niacin (µg)	67.08	36.24	13.0	-
Folic acid (µg)	0.98	0.94	0.22	-
Pantothenic acid (µg)	28.66	-	3.60	-
Biotin (µg)	0.28	0.14	0.02	-
Vitamin C (mg)	0.54	1.08	0.00	-
Choline (mg)	1.46	-	-	-
Inositol (mg)	0.30	-	-	-
Minerals				
Calcium (mg)	5.00	5.80	1.00	-
Phosphorus (mg)	2.68	3.62	0.92	-
Magnesium (mg)	0.36	0.48	0.16	-
Iron (mg)	0.12	0.08	0.00	-
Zinc (mg)	0.04	0.06	0.00	-
Manganese (µg)	0.30	-	1.60	-
Copper (µg)	5.80	3.62	0.60	-
Iodine (µg)	0.36	0.86	0.10	-
Selenium (µg)	0.12	0.10	0.02	-
Sodium (mg)	1.52	2.02	0.40	-
Potassium (mg)	6.70	6.52	1.46	-
Chloride (mg)	4.14	3.04	0.84	-

Data shown as per gram.

† Nutrient from the manufacturer

‡ Nutrient calculated from Nutritics software

Table S2. All significantly different species between day 7 and day 90 samples at baseline (T = 0 h).

Species (feature)	Coef	stderr	N	pval	qval
Escherichia_coli	-5.19026	0.993928	24	3.08E-05	0.00148
Blautia_wexlerae	3.995032	0.895915	24	0.000197	0.004717
Faecalibacterium_prausnitzii	5.928033	1.638332	24	0.001523	0.013367
Anaerostipes_hadrus	4.467974	1.22383	24	0.001408	0.013367
Faecalibacillus_intestinalis	1.649832	0.448441	24	0.001315	0.013367
Collinsella_aerofaciens	2.721403	0.760212	24	0.001671	0.013367
Streptococcus_parasanguinis	3.113727	0.906114	24	0.002357	0.016165
Sarcina_ventriculi	4.925132	1.522609	24	0.003808	0.022846
Intestinibacter_bartlettii	4.074808	1.294591	24	0.004674	0.024929
Dorea_longicatena	3.332809	1.077527	24	0.005311	0.025493
Schaalia_SGB17153	1.879816	0.63967	24	0.007598	0.033154
Granulicatella_SGB8255	2.961645	1.041372	24	0.009441	0.035085
Clostridiales_bacterium_KLE1615	2.663371	0.943677	24	0.009919	0.035085
Veillonella_atypica	3.503426	1.260667	24	0.010944	0.035085
Streptococcus_infantis	1.239562	0.446171	24	0.010964	0.035085
Streptococcus_salivarius	4.848445	1.765639	24	0.011792	0.035377
Segatella_copri	1.864647	0.688765	24	0.012868	0.036333
Romboutsia_timonensis	1.853047	0.722547	24	0.017675	0.047134
Ruminococcus_sp_AF13_28	2.044888	0.807761	24	0.019008	0.04802
Dorea_formicigenerans	2.209428	0.894393	24	0.021728	0.049665
Veillonella_dispar	1.390883	0.558631	24	0.020825	0.049665

Table S3. All significantly different species between day 7 and day 90 samples after 36 h of *in vitro* fermentation in the presence of inulin.

Species (feature)	Coef	stderr	N	pval	qval
Streptococcus_parasanguinis	2.986689	0.915813	24	0.003576	0.048077
Blautia_wexlerae	3.538593	1.101302	24	0.004006	0.048077

Table S4. All significantly different CAZyme families between day 7 and day 90 samples at baseline (T = 0 h).

Family(feature)	Coef	stderr	N	pval	qval
GH13_39	3.950903	0.184568	24	3.21E-16	6.70E-14
GH120	3.625112	0.312444	24	7.57E-11	7.91E-09
GH13_4	4.870421	0.541989	24	8.13E-09	5.67E-07
GH43_4	3.096174	0.408611	24	1.44E-07	7.50E-06
CBM13	3.765083	0.584648	24	1.77E-06	4.10E-05
GH43_12	2.861691	0.427288	24	9.87E-07	4.10E-05
CE7	4.23399	0.654771	24	1.66E-06	4.10E-05
GH27	3.911072	0.594298	24	1.28E-06	4.10E-05
GH31_15	3.447489	0.530962	24	1.57E-06	4.10E-05
GT56	-5.03641	0.802282	24	2.56E-06	5.36E-05
GH37	-4.48685	0.767414	24	6.98E-06	0.000133
GH103	-4.4418	0.775593	24	9.25E-06	0.000153
GH13_19	-4.15755	0.727365	24	9.49E-06	0.000153
GH3	1.009526	0.182814	24	1.50E-05	0.000224
CE20	2.687782	0.529858	24	4.42E-05	0.000616
GH95	1.99376	0.397024	24	5.00E-05	0.000653
AA1	-3.93697	0.802031	24	6.57E-05	0.000808
AA6	-4.37727	0.908781	24	8.22E-05	0.000859
GT20	-3.87949	0.802184	24	7.84E-05	0.000859
GH13_14	3.19463	0.659197	24	7.65E-05	0.000859
GH20	3.459967	0.73844	24	0.000113	0.001115
GH31_4	2.848872	0.609994	24	0.000117	0.001115
GH31_13	-3.884	0.86037	24	0.000172	0.001561
GH102	-3.64255	0.821347	24	0.000209	0.001816
GH140	3.993302	0.910235	24	0.000234	0.001959
GH130_1	1.940347	0.447823	24	0.000268	0.002148
GH31_5	2.928167	0.678122	24	0.000277	0.002148
GH24	-4.68507	1.091824	24	0.000296	0.002212
GT9	-4.33511	1.033403	24	0.000375	0.002701
GH130_2	2.464461	0.593195	24	0.000414	0.002882
GH36	1.463723	0.353521	24	0.000428	0.002887
CBM32	3.630514	0.885757	24	0.000474	0.003096
GH31_1	3.015449	0.739877	24	0.000502	0.003177
GH127	2.456595	0.615814	24	0.000619	0.003807
GH13_31	2.950377	0.746859	24	0.000681	0.004064
GH42	3.028472	0.773433	24	0.000741	0.004206
GT101	2.585029	0.660543	24	0.000745	0.004206
GH43_27	2.424493	0.623729	24	0.000794	0.004255
GH43_24	1.821363	0.467778	24	0.000781	0.004255
GH23	-1.87073	0.484546	24	0.000846	0.004423
CE2	2.513807	0.65339	24	0.000875	0.004458
GH133	2.012067	0.525438	24	0.000914	0.004547
GH32	1.373529	0.364054	24	0.001048	0.005093
GH13_21	-3.54723	0.946548	24	0.001114	0.005292

GH153	-3.51924	0.959206	24	0.001348	0.006259
GH13_20	2.636625	0.732455	24	0.001593	0.007236
GH43_35	2.705191	0.758623	24	0.001728	0.007551
GH13_27	-3.71624	1.04262	24	0.001734	0.007551
CE14	-3.50409	0.986163	24	0.001781	0.007597
CE17	1.56448	0.450527	24	0.002162	0.009036
GH91	1.659153	0.481339	24	0.002298	0.009419
PL11	1.526609	0.444517	24	0.002369	0.009521
GH13_8	1.03091	0.301908	24	0.002483	0.009791
GT2	0.513677	0.151621	24	0.002647	0.010243
GH123	2.072201	0.618196	24	0.002883	0.010809
CBM66	2.310056	0.689551	24	0.002896	0.010809
GH106	1.949795	0.584312	24	0.002988	0.010957
GH5_44	2.4462	0.762674	24	0.004061	0.014632
GH18	2.951469	0.924055	24	0.00419	0.014844
CBM91	1.879211	0.591428	24	0.004358	0.014923
CE6	1.968229	0.620754	24	0.004427	0.014923
GH30_1	1.297164	0.408503	24	0.004378	0.014923
GH63	-3.10155	0.983995	24	0.004626	0.015346
GH112	2.776965	0.886062	24	0.004825	0.015756
GH10	0.932987	0.298597	24	0.004933	0.015862
GT108	0.917569	0.297296	24	0.005394	0.017081
GT51	-0.80799	0.263744	24	0.005689	0.017747
CE8	-2.20094	0.720211	24	0.005791	0.017797
PL33_1	0.792938	0.262976	24	0.006366	0.018738
GH30_3	1.104056	0.364837	24	0.006206	0.018738
CBM6	0.92348	0.306136	24	0.006346	0.018738
GT32	2.260758	0.755779	24	0.006729	0.019533
GT30	-2.84093	0.965297	24	0.007522	0.021074
GH146	1.89282	0.643156	24	0.007523	0.021074
GH16_3	1.638876	0.557297	24	0.007562	0.021074
GH51_2	1.764817	0.60472	24	0.007962	0.02161
GH13_44	2.535615	0.867877	24	0.007903	0.02161
CBM51	1.084716	0.375205	24	0.008479	0.022719
GH84	1.308664	0.460275	24	0.009458	0.024403
GH43_10	1.84928	0.649262	24	0.009349	0.024403
GT6	1.761343	0.618896	24	0.009399	0.024403
CE11	-3.15276	1.117419	24	0.009938	0.025331
GH13_36	1.281054	0.45675	24	0.010324	0.025998
GH29	1.526309	0.56355	24	0.012835	0.031339
GH5_2	1.780326	0.657849	24	0.012895	0.031339
GT39	1.682087	0.619507	24	0.01264	0.031339
GH13_9	1.959451	0.727892	24	0.013317	0.031991
GH43_17	1.325086	0.503329	24	0.015203	0.036107
GH67	0.686844	0.262309	24	0.01569	0.036154
PL11_1	0.940728	0.359268	24	0.01569	0.036154
GT81	1.663491	0.635654	24	0.015742	0.036154

GH8	-2.1091	0.8096	24	0.016161	0.036319
GH43_31	0.792427	0.303747	24	0.016029	0.036319
PL26	0.555575	0.214509	24	0.016712	0.037157
PL12_3	0.98631	0.382744	24	0.017201	0.037841
GT83	-2.62897	1.025489	24	0.017713	0.038563
CBM35inCE17	1.165182	0.459313	24	0.018791	0.040487
GT111	2.140406	0.849915	24	0.019565	0.041356
GT5	1.803728	0.716902	24	0.019667	0.041356
CBM57	1.330425	0.529373	24	0.019787	0.041356
GH172	1.391149	0.557606	24	0.020597	0.042535
GH165	1.155578	0.463851	24	0.020759	0.042535
SLH	1.648138	0.66456	24	0.021273	0.043166
CBM97	0.986473	0.400088	24	0.02195	0.044111
GH51_1	2.035375	0.829378	24	0.022507	0.044417
GH35	2.142667	0.873248	24	0.022527	0.044417
GH182	1.141795	0.468897	24	0.023454	0.045812
GT73	-2.07914	0.855969	24	0.023764	0.045987
GH78	2.736079	1.139703	24	0.025258	0.047991
GH50	0.780423	0.32478	24	0.025137	0.047991
